# Management of an Eroded Gastric Ring Following the Third Metabolic Bariatric Surgery: A Multimedia Article

**DOI:** 10.1007/s11695-025-07702-1

**Published:** 2025-01-30

**Authors:** Mohamed Hany, Anwar Ashraf Abouelnasr, Mohamed Ibrahim, Ahmed Elshamarka, Asmaa Hamdy, Bart Torensma

**Affiliations:** 1 Medical Research Institute, Alexandria University, Alexandria, Egypt; 2 Leiden University Medical Center, Leiden, the Netherlands

**Keywords:** Metabolic bariatric surgery, Eroded gastric, Band, Ring, Revision surgery, Ring-augmented Roux en Y gastric bypass, Roux en Y gastric bypass

## Introduction

Revisional surgery is an expanding subset of MBS, growing in necessity as primary surgeries increase. A systematic review of primary MBS highlighted inconsistencies in reporting reasons for suboptimal clinical responses. Many studies on revisional operations lack standardized selection criteria. The most common definition of suboptimal clinical response was less than 50% excess weight loss (EWL) and/or a body mass index (BMI) > 35 kg/m^2^ at 18 months postoperation. Other indications for revisional surgery include dysphagia and gastroesophageal reflux disease (GERD) [1].

One such approach, ring-augmented Roux-en-Y gastric bypass (rRYGB), has demonstrated more significant weight loss than standard RYGB but remains controversial due to potential complications associated with the silicone ring. While studies report low rates of ring-related complications (0–4%), some surgeons exercise caution [2–4].

The hypothesized mechanism of action is that the ring induces earlier satiety by exerting pressure on the gastric wall when the pouch is full without causing restriction when the pouch is empty.

Several publications report 5-year follow-up outcomes for rRYGB and non-rRYGB patients, with higher weight loss observed in the rRYGB group and similar complication rates across both groups [2, 3]. However, studies describing outcomes beyond 5 years are limited, often featuring low follow-up rates, which may render the findings less representative [5].

Dapri et al. described results from six patients undergoing non-adjustable silicone ring placement after suboptimal weight loss following RYGB. These patients achieved an average percentage EWL of 70% 1-year post-revision, though their average preoperative BMI was 29.5 kg/m^2^ [6]. Despite promising outcomes, rRYGB is not without risks, the most notable being ring erosion, with reported long-term erosion rates between 0 and 2% [7, 8]. Eroded rings are typically removable endoscopically, though some cases may require surgical intervention.

We present a case of a patient treated at our clinic who underwent a ring-augmented RYGB as a tertiary surgical intervention. This patient had previously experienced weight recurrence following an initial LSG and a subsequent RYGB performed at an external center. The complexity of this case was compounded by early ring erosion following the most recent procedure.

### Case Presentation

In 2014, a 37-year-old female patient underwent an LSG as her initial MBS procedure. At the time of the LSG, she weighed 122 kg, had a height of 165 cm, and had a BMI of 44.8 kg/m^2^. This intervention successfully resulted in a 51-kg (kg) weight reduction, bringing her to 71 kg. However, by 2018, the patient experienced significant recurrent weight gain, reaching 103 kg and presented with symptoms of GERD. Consequently, she underwent RYGB to manage her weight and reflux symptoms. While the initial LSG was performed at our center, the subsequent RYGB was conducted at another facility with limited operative data.

Following the RYGB, the patient lost 12 kg, falling short of her weight loss goals. Imaging studies, including virtual 3D CT volumetry, revealed a gastric pouch volume of 200 cc, while upper gastrointestinal (GI) endoscopy identified a long blind candy cane limb. A multidisciplinary evaluation presented the patient with several surgical options discussed in detail, including associated risks and potential intraoperative modifications.

The surgical options included resizing the gastric pouch and/or gastrojejunostomy, performing distalization, and adding ring augmentation around the pouch. A duodenal switch (DS) was also considered if sufficient remnant antrum allowed sleeve pouch reconstruction, either as a one- or two-stage intervention. After careful deliberation, the patient resized the stoma, distalizing the small bowel, and placed a ring.

In June 2023, surgery commenced with adhesiolysis, followed by resizing the pouch and gastrojejunal stoma over a 40 Fr bougie. Intestinal limb lengths were evaluated: an alimentary limb (AL) of 70 cm, a biliary limb (BL) of 60 cm, and a common channel (CC) of 550 cm. The BL was extended to 260 cm, and the CC was shortened to 350 cm. A minimizer ring (Bariatric Solutions, Switzerland) was loosely placed around the gastric pouch and secured with non-absorbable sutures. The procedure lasted 110 min, and the patient was discharged on postoperative day two.

### Postoperative Course

In the initial postoperative period, the patient achieved a 12-kg weight loss within 2 months. However, by the third month, she developed progressive dysphagia, reduced food tolerance, and difficulty consuming her usual dietary intake. Despite proton pump inhibitor (PPI) therapy and nutritional modifications, her symptoms persisted.

Persisting symptoms led to a CT scan demonstrating a standard contrast passage with slight fat stranding around the stomach. Subsequent upper GI endoscopy revealed partial ring erosion and associated ulceration on the gastric wall, although the gastrojejunostomy remained patent. Attempts at endoscopic removal of the eroded ring were unsuccessful.

Adhesiolysis was performed to mobilize and remove the ring. Sutures were placed at the erosion site, and an omental patch was applied for reinforcement. The procedure lasted 40 min, and the patient was discharged on postoperative day two after tolerating oral fluids. A CT scan with oral contrast showed no complications.

The patient was prescribed PPIs and sucralfate for six months. By September 2024, her symptoms had resolved, and she achieved a total weight loss of 27 kg, reducing her weight to 76 kg and her BMI to 27.9 kg/m^2^.

## Conclusion

Patients presenting with upper gastrointestinal symptoms after ring-augmented RYGB should undergo a thorough evaluation, especially when symptoms persist despite medical management. In cases of ring erosion into the gastric pouch, a combined laparoscopic and endoscopic approach offers a viable and effective method for ring removal.

## Supplementary Information

Below is the link to the electronic supplementary material.

Supplementary file1 (MP4 322850 KB)

## Data Availability

No datasets were generated or analysed during the current study.
